# Evaluating the Effects of Capacity Building Initiatives and Primary Care Networks in Singapore: Outcome Harvesting of System Changes to Chronic Disease Care Delivery

**DOI:** 10.3390/ijerph20032192

**Published:** 2023-01-25

**Authors:** Andrew Teik Hong Chen, Gerald Choon-Huat Koh, Ngan Phoon Fong, Jeremy Fung Yen Lim, Zoe Jane-Lara Hildon

**Affiliations:** 1 Saw Swee Hock School of Public Health, National University of Singapore, and National University Health Systems, Singapore 117549, Singapore; 2 Future Primary Care, Ministry of Health Office of Healthcare Transformation (MOHT), Singapore 099253, Singapore

**Keywords:** outcome harvesting, PCN, chronic disease management, chronic disease registry, ancillary services, team-based care, Healthier SG

## Abstract

The high tertiary healthcare utilisation in Singapore due to an ageing population and increasing chronic disease load has resulted in the establishment of primary care networks (PCNs) for private general practitioners (GPs) to provide team-based, community care for chronic diseases. A total of 22 PCN leaders and programme managers from 10 PCNs participated in online group discussions and a survey. Outcome harvesting was used to retrospectively link the intended and unintended outcomes to the programme initiatives and intermediate results (IRs). The outcomes were generated, refined and verified before shortlisting for analysis. About 134 positive and 22 negative PCN outcomes were observed since inception in 2018. By establishing PCN headquarters and entrusting PCN leaders with the autonomy to run these, as well as focusing policy direction on GP onboarding, GP engagements and clinical governance, the programme successfully harnessed the collective capabilities of GPs. Developments in the organisation (IR1) and monitoring and evaluation (IR4) were the top two contributors for positive and negative outcomes. Sustainable practice and policy changes represented 46% and 20% of the positive outcomes respectively. Sustainable positive outcomes were predominantly contributed by funding, clear programme policy direction and oversight. Conversely, most negative outcomes were due to the limited programme oversight especially in areas not covered by the programme policy.

## 1. Introduction

The ageing population and rising prevalence of chronic diseases in Singapore have resulted in an unparalleled burden on the hospital-centric health system [1,2]. The national government healthcare expenditure of the current hospital-centric care model in Singapore is not sustainable and has been projected to triple to almost $27 billion in 2030 [3].

Many healthcare systems rely on strong primary and preventive care systems to contain healthcare utilisation and cost [4]. Singapore has been perceived by primary care experts to have a weak primary care system [5]. For instance, long-term care for chronic diseases has largely still been managed in the overcrowded hospital specialist outpatient clinics (SOCs) [6].

The primary care ecosystem in Singapore can be divided into the private general practitioner (GP) clinics and the polyclinics [7]. The majority of the 1800 private GP clinics are single physician practices with limited support and capacity to provide ancillary services such as diabetic retinal photography, diabetic foot screening and nurse counselling [8,9]. In contrast, the government-funded polyclinics are multi-doctor clinics, self-sufficient with diagnostic and ancillary services to provide team-based care at heavily subsidised rates [7,10]. With private GPs making up 80% of the total workforce in the sector and managing a disproportionately lower chronic disease workload of 59% collectively, the strain on the polyclinics and SOCs has compelled right-siting of care to the private GPs [11].

In order to strengthen the primary care system, Singapore’s Ministry of Health (MOH) sought various avenues, notably through primary care networks (PCNs) in which the collective capabilities of the private GPs could be harnessed. As an individual organisational entity, each PCN is led by two GP leaders with the support of a team of nurses, primary care coordinators and administrators [12]. Such team-based networks facilitate resource sharing amongst its GPs, needed to scale optimal chronic disease care and rein in escalating cost of healthcare [13]. Similar inter-professional networks in Canada and New Zealand have also augmented the delivery of integrated primary care and chronic disease care [14,15].

The PCN development was steered through funding and policy direction set out by MOH and administered by the PCN Oversight Agency (POA), the Agency of Integrated Care (AIC). The POA provided oversight over PCN growth areas such as GP onboarding, PCN GP upskilling and clinical governance. In addition, POA centralised procedures by standardising processes and training for the networks. MOH and POA were responsible for the creation and administration of the monitoring and evaluation (M&E) systems using a chronic disease registry (CDR) and the Care Plus Fee pay-for-performance framework, which will be further elaborated in the results section.

### 1.1. Conceptual Model

Intermediate results (IRs) refer to the key areas and related outputs from the programme initiatives. The PCN programme was therefore built around the implementation of these IRs. As such, the IRs connect the initiatives undertaken by MOH and POA needed to catalyse change to the harvested PCN outcomes. In addition, as the pre-cursors to the outcomes, the IRs also serve to organise the structure and the content of the analysis.

**Figure 1:**
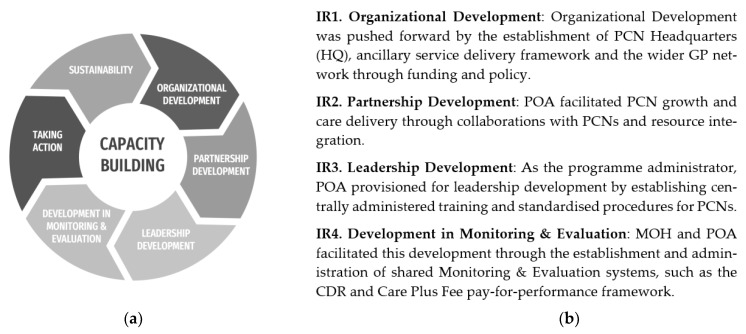
(**a**) Capacity building model adapted from Centre for Innovative Education Solutions (CIES). (**b**) Outline of the four IRs.

The IRs are theoretically informed and are anchored to the capacity building model domains as defined in Figure 1, following the approach advanced by Centre for Innovative Education Solutions [16]. The four IRs are outlined accordingly.

### 1.2. Aims and Research Questions

This study, which follows the implementation science tradition [17], aims to evaluate and systematically assess the wide-ranging outcomes independently achieved by the PCN programme participants. The evaluation is being conducted close to the five-year milestone of the PCN programme, hence, this study is able to provide timely insights as Singapore prepares to shift towards a national population health approach through ‘Healthier SG’ [18].

In order to achieve the evaluation aims, the study is guided by the following questions:

1. What are the observed PCN outcomes that have resulted from the capacity building initiatives of the programme?
2. How sustainable are the outcomes that have been initiated by participation in the PCN?

## 2. Methods

### 2.1. Methodology Overview

MOH, POA and PCNs have continued to innovate and adapt the capacity building approach during the programme. So, the dynamic programmatic changes have constrained the use of the more conventional evaluation methods that compare pre- and post-assessments. Due to the complexity of the programme, the prospective outcomes that would result from the inputs and interventions introduced by MOH and POA were not known a priori.

Outcome harvesting is a qualitative method of data elicitation and was used to evaluate the intended and unintended PCN practice changes that were influenced by the programme contributions. As a complexity-aware methodology, outcome harvesting was used to retrospectively generate and analyse the collated outcomes, which the programme owners and evaluators had difficulties predicting and determining at the outset of the intervention. All outcomes were treated equally, and recorded whether positive or negative. Each outcome had to be linked to the programme intermediate results and yet needed to be an independent achievement driven by the PCN members, who were trained or supported by the programme [19].

**Figure 2:**
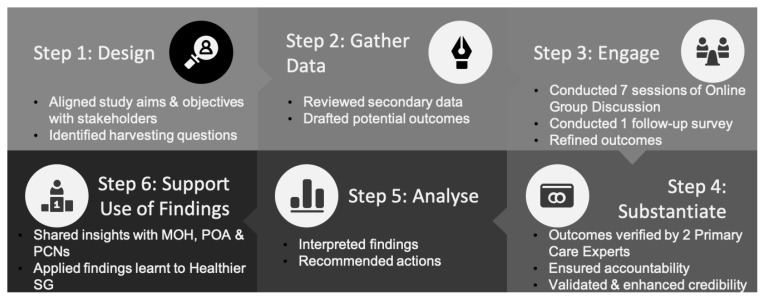
Diagrammatic representation of outcome harvesting six step process.

The evaluation was conducted by adhering to the six sequential steps of outcome harvesting [20,21] (Figure 2).

After research questions were identified and context understood by reviewing programme documentation, PCN leaders and programme managers were engaged, and seven sessions were undertaken to generate discussion around each of the four IRs. Sampling for these was strategic and purposive. PCN GP leaders, programme managers and administrators were chosen because they were considered to be able to provide the most accurate, first-hand insights on the effects of the programme initiatives and how their own practices had changed through these. The clinical leaders helm the PCN. Both the clinical leaders and the administrative leaders oversee the strategic planning, management and operations of the PCNs. The programme managers implement the daily operations.

A total of 22 participants took part in the online discussion sessions, as described below. Types of independently achieved outcomes were elicited around each of the IRs, and these were recorded with the dates of implementation and type of capacity building initiative tagged to them. A REDCap survey was then used to follow-up with these respondents in case additional outcomes were remembered post-hoc. The survey responses were integrated into the list of outcomes generated from the discussion groups, serving both to confirm and extend the list. The outcomes were then further refined through verification and cross-checking with key stakeholders before finalising and shortlisting all agreed outcomes for analysis.

Outcome harvesting is interpreted using a mix of descriptive counts and narrative interpretation; no statistical tests are required. The unit of analysis are the outcomes achieved.

### 2.2. Ethical Procedures

Ethics approval (SSHSPH-151) was obtained from Saw Swee Hock School of Public Health Department Ethics Review Committee (SSHSPH-DERC) before starting the study.

### 2.3. Participant Profile

A core sample of six clinical leaders, five administrative leaders, nine programme managers, and two project executives were recruited and represented regional and organisational Primary Care networks. Each of the 22 participants from ten out of eleven PCNs took part in an online facilitated group discussion and a follow-up survey. Another administrative leader who was not able to participate was represented by the two project executives.

## 3. Results

### 3.1. Question 1: What Are the Observed PCN Outcomes That Have Resulted from the Capacity Building Initiatives of the Programme?

**Figure 3:**
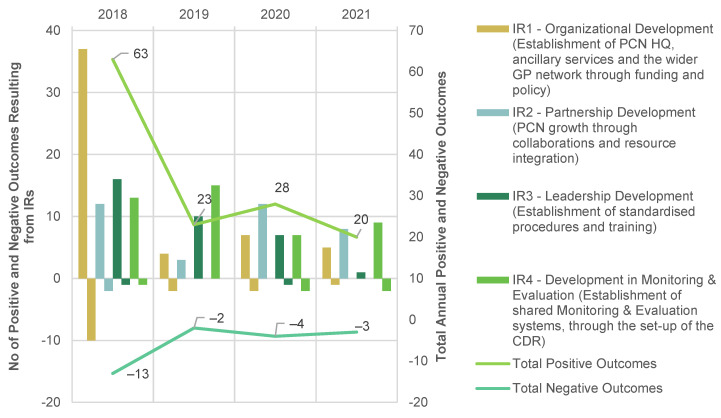
Positive (*N* = 134) and negative (*N* = 22) outcomes ^a^ tied to intermediate results (IRs) over time ^b^. ^a^ Summary table of the positive and negative outcomes over time can be viewed in Table A1, in Appendix A. ^b^ Some outcomes have been contributed by more than one IRs over time. Hence the sum of all four IR counts per year exceeded the annual total count.

There were altogether 134 positive and 22 negative PCN outcomes observed as a result of the various IRs and related capacity building initiatives implemented through the programme, summarised in Figure 3 (see further breakdowns in Table A1 in Appendix A). Positive outcomes were achieved when the programme IRs evolved intentionally to improve system dynamics. The negative outcomes are classified as outcomes that were not able to achieve their original intent, i.e., those with difficulties scaling and outcomes which had the potential for negative implications.

Amongst the outcomes achieved as a result of the four IRs between the inception of PCN in 2018 and the start of the evaluation in November 2021, almost half the positive outcomes were either initiated or achieved in 2018 and predominantly related to organisational development (IR1). A smaller peak of 28 positive outcomes occurred in 2020, seven of which were related to PCN’s response to COVID-19.

The trend for the annual negative outcomes mirrored that of the positive outcomes over time. Generally, all four IRs had generated substantive positive outcomes, indicating a good implementation efforts and follow through. The top two IRs were IR1, pertaining to Organisational Development and IR4 relating to Development of Monitoring and Evaluation. These achieved 40% and 33% of positive outcomes respectively. The negative outcomes mirrored the trend demonstrated by the positive outcomes and they predominantly resulted from challenges in organisational development.

Aside from linking the outcomes to capacity-building IRs, the outcomes were alternatively classifiable into thematic outcome categories (1–8). These are described briefly in turn and then related to IRs.

Starting with *Category 1 Network Development*, these outcomes relate to anything initiated by each PCN HQ to grow the GP network e.g., GP onboarding, GP engagement and corporate governance. *Category 2 Service Provision*, consists of new clinical services provided by the PCN to augment primary health care, which includes chronic disease management. Outcomes in *Category 3 Clinical Practice* and *Category 4 Care Delivery* refer to initiatives that improved clinical outcomes and processes respectively. Outcomes relating to the set-up of a framework for monitoring and evaluation are grouped under *Category 5 Benchmark and Standards. Category 6* relates to *Resource Sharing*, typified by the outcomes arising from inter-PCN and external collaborations. *Category 7* consists of *Harnessing Enablers*, for example IT and financial enablers that facilitate PCN in achieving its objectives. Finally, *Category 8* records *learning*-related activities as initiated by and for PCN GPs and extended staff.

**Figure 4:**
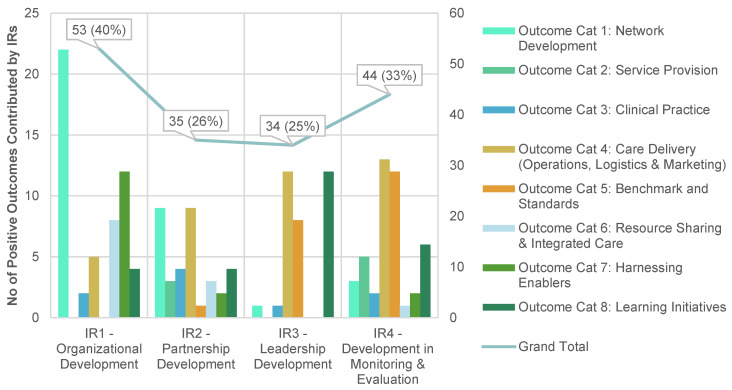
Positive outcomes (*N*= 134) ^a^ contributed by intermediate results (IRs) ^b^. ^a^ Please refer to breakdown of positive outcomes according to categories in Table A2 in Appendix A. ^b^ Some outcomes have been contributed by more than one IRs, so the sum of percentages have exceeded 100 percent.

**Figure 5:**
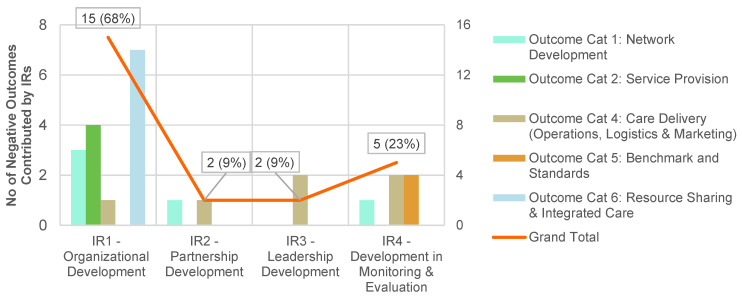
Negative outcomes (*N* = 22) ^a^ contributed by intermediate results (IRs) ^b^. ^a^ Please refer to breakdown of negative outcomes according to categories in Table A3 in Appendix A. ^b^ Some outcomes have been contributed by more than one IRs, so the sum of percentages have exceeded 100 percent.

Positive outcomes are summarised in Figure 4, and negative ones in Figure 5 (see further breakdowns in Table A2 and Table A3 respectively in Appendix A).

The eight thematic outcome categories also served to guide a deeper regrouping and exploration derived from both the positive and negative (*N* = 156) total harvested outcomes. Grouped outcomes are listed by outcome category in Table A4 in Appendix B, and are narrated in turn below.

#### 3.1.1. IR1 Organisational Development: Establishment of PCN HQ, Ancillary Services and the Wider GP Network through Funding and Policy

From the programme outset, MOH recruited and remunerated GP leaders to undertake the role of a clinical leader and administrative leader for each PCN. Using MOH funds, the PCN leaders were able to hire key roles needed to establish the PCN headquarters and provide ancillary services. The organisational development was also guided by the programme policy which focused on specific growth areas such as GP onboarding and chronic disease patient load.

By funding and focusing the programme policy on specific key growth areas, MOH was able to direct PCNs’ organisational development. In addition, the policy was designed to be less prescriptive, thereby entrusting PCN HQs and some parent organisations with the capacity and autonomy for governance, strategic planning and operations. For instance, by allocating its PCN HQ staff with similar portfolios within a Healthcare Cluster, the parent organisation in the Healthcare Cluster was able to facilitate the operations of both entities.

PCNs have the capacity to develop teams comprising nurse counsellors and primary care coordinators in order to deliver chronic disease ancillary services such as diabetic retinal photography, foot screening and nurse counselling. Some PCNs deployed ancillary services during non-clinic hours, rotated teams between the clinics or trained clinic staff in providing these services. The one-stop convenience for the patients have been perceived to significantly reduce the barriers to service utilisation.

Five PCNs opted to develop organic capabilities for ancillary services, which was perceived to provide more flexibility than the use of external providers. The in-house model has also enabled two PCNs to decentralise ancillary service delivery using two or more teams. The remaining PCNs had decided against developing in-house capabilities due to the limited subvention available to maintain more than one such team, capital-intensive DRP machines and large GP networks to service. 

Sixty-eight percent of all the negative outcomes were due to IR1. First, the limited programme oversight in certain areas of PCN operations might have contributed to the lack of alignment for GP catchment policies and initiatives for decanting simpler cases to private GPs. Second, the functional areas of business management in the PCN HQs such as corporate governance, marketing, finance and IT, have remained relatively under-developed.

As the overall funding framework was determined based on the projections at the start of the programme, there was no visibility for future areas of need or evolving challenges. Whilst piece-rate subvention remained available, the requests for funding would need to be aligned with the growth areas stipulated by the programme. Hence, there was a general lack in subvention for operations that did not constitute specific planned growth areas of the programme. Nevertheless, this has encouraged some PCNs to innovate or seek other funding sources.

#### 3.1.2. IR2 Partnership Development: PCN Growth through Collaborations and Resource Integration

POA oversees the disbursement of MOH funds to the PCNs. This has helped POA administer the programme by collaborating closely with the PCN HQs in areas of growth and resource integration. For instance, POA steered collaborations in line with the MOH policy direction during the quarterly PCN Council meetings and by introducing external stakeholders. This has successfully seeded three MOH Office for Healthcare Transformation (MOHT) GP Innovative Initiatives amongst the PCNs. These PCNs were well supported financially, thereby contributing to the remarkable progress and effectiveness of the initiatives.

The nine (25%) IR2 outcomes that resulted in network development highlighted the critical role that POA played in catalysing cohesion and engagements within PCNs. The regular PCN team-based activities and communication involving GPs have resulted in peer-led collaborative learning and sharing of best practices.

POA also coordinated, rallied and supported frontline GPs, thereby contributing significantly to the expedient and effective COVID-19 responses. In fact, the social capital within the networks that had developed as a result of the ongoing engagements was key to the swift implementation of COVID-19 measures demonstrated by the PCNs. Its pandemic responses are reflected in eight (23%) positive IR2-related outcomes.

#### 3.1.3. IR3 Leadership Development: Establishment of Standardised Procedures and Training

In order to oversee the growth areas and policy direction set out by MOH, POA also established centrally administered training and standardised PCN procedures. These influenced positive outcomes in learning initiatives which were independently taken forward by the PCNs. These trainings and sharing of procedures led to application of benchmarks and standards driving better performance and care delivery. The achievements of IR3 therefore mostly related to centralising of training for PCN HQ and clinic staff, ultimately enabling better understanding for quality improvement and leadership relating to operations, logistics and marketing.

Though 25% of outcomes were reached for IR3 in the above narrated categories, there were minimal to no positive outcomes in other outcome categories as these did not align well with the growth areas for IR3 as defined by POA.

#### 3.1.4. IR4 Development in Monitoring and Evaluation: Establishment and Administration of Shared Monitoring and Evaluation Systems

Various programme levers have contributed to the successful development in monitoring and evaluation: MOH policy direction in clinical governance; the set up and maintenance of the CDR by POA; and the pay-for-performance framework using Care Plus Fee funding to incentivise good clinical governance. MOHT and some parent organisations have also contributed to the development in monitoring and evaluation by aligning IT systems and automating processes within some PCNs.

The CDR is a digital registry utilised as the main framework for benchmarking chronic disease care delivery standards in PCNs. It contains PCN patient data ranging from sociodemographic profiles, process and clinical indicators in chronic disease management. Submissions of relevant CDR data to POA have encouraged GPs to manage patients according to the clinical practice guidelines embedded within the framework of the CDR. In return, GPs receive a Care Plus Fee based on the quantum of $100 for every chronic disease patient who has been successfully managed and notified through the CDR. In addition, the data were also analysed and used to benchmark the performance of the PCNs and their clinics.

The use of Macro Excel as the main platform for the CDR means that data has to be manually maintained at each level to ensure data fidelity. Such data management processes pose significant administrative burden for GPs and their clinic staff despite the support that has been rendered by programme managers and primary care coordinators. Automation afforded by the in-house electronic medical records and data extraction capabilities in some PCNs have also been limited in alleviating the administrative workload.

Despite the challenges, the CDR has been a useful monitoring tool. Altogether, IR4 contributed 33% of the overall positive outcomes. The regular performance benchmarking using the CDR has encouraged twice-yearly gap analyses and developed in the GPs a sense of ownership for the data and for quality improvement initiatives. For instance, some PCNs have developed dashboards for easy visualisation of clinic performances. Moreover, IR4 has resulted in various innovative interventions through automation and streamlining of manpower and operations to mitigate the administrative burden caused by the CDR.

### 3.2. Question 2: How Sustainable Are the Outcomes That Have Been Initiated by Participation in the PCN?

In the second evaluation objective, the sustainability of each outcome was assessed based on whether the outcome was sustained for at least six months or repeated over the course of the programme. Sustained practice change was demonstrated at the individual, institutional or systemic level. When an institutional or systemic policy change was demonstrated, the outcome was classified as sustained. The sustainability of outcomes was self-reported based on sustainability-related questions embedded in the outcome harvesting tool and follow-up survey.

**Figure 6:**
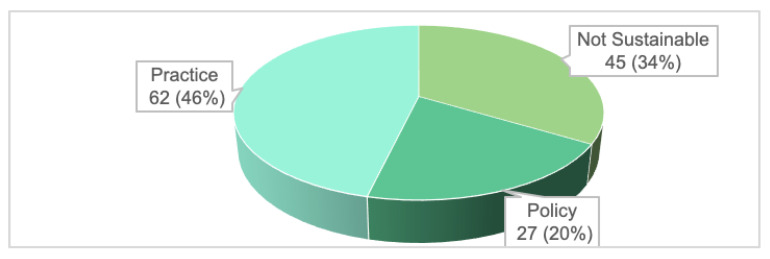
Sustainability of positive outcomes (*N* = 134).

Out of a total of 134 positive outcomes, 89 (66%) were identified as sustainable outcomes, see Figure 6. Sixty-two of these (46%) represented sustainable practice changes, and 27 (20%) were related to sustainable policy changes. The sustainable practices and policies observed in the PCNs generally were connected to growth areas stipulated by the programme. This was indicative of the success of the long-term vision and planning embedded in the PCN approach and of the chosen IRs.

The 62 sustainable PCN practices were demonstrated in various aspects of PCN operations such as structured engagements, ancillary service delivery, ground-up clinical initiatives, nurse counselling practices, data management, gap analyses and training. The 27 sustainable policy outcomes were broadly grouped in the following areas: governance, structured engagement and GP support; clustering of ancillary service delivery; Family Medicine Clinic (FMC) right-siting initiatives; and automation through IT systems. There was a notable negative PCN practice where the internal referral system to tertiary care within the same organisation had resulted in the under-development of mental health care delivery amongst its PCN GPs, which would be crystalised as part of institutional practice, unless reversed.

## 4. Discussion

‘Healthier Singapore’ is a population health initiative to promote healthier lifestyles in order to improve the quality of life of its residents and reduce the rising chronic disease burden. This is achieved by anchoring each Singapore resident with a GP and fostering community support for healthier lifestyles [22].

Based on the wide-ranging outcomes harvested in this evaluation, the PCNs have successfully fulfilled most of the programme aims and objectives, over and beyond the recommendations of the original PCN pilot [23]. However, as the PCNs transition into the Healthier SG national plan that aims to involve most of the 1800 GP clinics, the programme has to continue to scale-up [11]. The Healthier SG programme aims and objectives based on a nationwide reach, adoption, effectiveness and other contextual factors beyond 2022 will define the new framework for scaling through resource allocation, capacity building, policy development and addressing barriers [24].

The existing management and operations will not be sustainable as the PCNs expand significantly. Hence, the expansion plan will need to factor in the effect of growth and decentralisation [25]. For instance, a 40-clinic PCN had to decentralise further from the original two to three clusters of 12–15 clinics each in order to more effectively provide ancillary services. As such, effective access and utilisation of services will need to be considered in tandem with PCN expansion plans.

The increasing organisational complexity also necessitates the development of hierarchical structures and additional capabilities, which makes organisational development resource intensive. For example, the incorporation of GP clinics onto a unified health informatics platform and the access to subsidised medications require substantial resources [26]. By centrally coordinating shared resources involving multisectoral stakeholders and by encouraging PCNs to collaborate further through Healthier SG rather than operate in silo, MOH can minimise duplication of efforts and streamline resources. For instance, MOH has rolled out various Healthier SG initiatives including IT Enablement Grant to nudge GPs to adopt a Healthier SG compatible electronic medical records systems [22].

A carefully balanced complement of programme levers in the policy development for Healthier SG is key to engaging the various stakeholders. MOH and POA have effectively utilised the four IRs and balanced the synergistic interplay between a ground-up approach and centralisation in the policy design and administration. For instance, by entrusting GP leaders with the means to establish and manage PCNs based on a clear policy direction, the programme was able to organise GPs into peer-led networks and equip GPs with the infrastructure and roadmap for team-based, chronic disease care [27]. The capacity of the programme in motivating GPs to augment their practices in order to achieve its objectives was a key strength of this programme.

The shift from a workload-based model to a capitation model in Singapore’s healthcare financing may be the catalyst needed to pivot healthcare clusters and the PCNs towards population health and enhance integrated care between them [28]. The outcome-based capitation model remunerates based on improved clinical outcomes through preventive care, thereby departing from the piece-rate remuneration for reactive treatments of ill health.

As Singapore augments its capabilities in data and analytics, this will increase the visibility needed for stakeholders to collaborate and to drill down to the specific data points of the patients [29]. In addition, an effective data infrastructure provides the monitoring and evaluation scaffold needed to effect change and alignment.

Similarly, a well-designed and implemented partnership development within an integrated framework between healthcare clusters and PCNs is a necessary step towards scaling [30]. However, structural differences, orientation towards fee-for-service and tension between top-down and ground-up approaches remain.

In order to align all the diverse elements in the Healthier SG ecosystem, MOH and POA play increasingly critical roles in providing centralised leadership and facilitating stakeholder engagements [31]. Right-siting initiatives to decant patients from hospitals/polyclinics to private GPs were useful examples to illustrate the complex interplay of factors amongst stakeholders leading to scaling successes or challenges [6,32,33,34]. As such, the programme levers for the partnership development should be based on building trust and mutual collaboration [31].

Legacy issues such as the financial gradient between private GP practices and polyclinics and the GP consultation fee structure can only be calibrated by involving the population as deeply entrenched public perceptions and health behaviours that dictate GP practices persist. COVID-19 has taught us that population behaviours can be shaped. However, this will require structural issues such as low GP consultation fees, GP clinic drug dispensing model and resource sharing in the purchase of medications to be addressed holistically and by engaging the public [35].

Finally, significant strides will also need to be made to connect the residents with the GPs. So, whilst empanelment was not on the agenda when the PCNs were designed, the trusted relationship between the resident and the doctor through empanelment is a critical enabler for the longer term [22,36,37]. In addition, coverage of the Healthier SG programme can be optimised through effective awareness campaigns to inform and educate the public.

### Strengths and Limitations

Outcome harvesting was effective in identifying wide-ranging organisational and systemic changes in capacity demonstrated through the PCNs. The methodology provided a systematic approach in the capture of both the intended and unintended outcomes, including positive and negative ones. Given that the approach treated all outcomes equally instead of focusing on planned change, outcome harvesting was useful especially in assessing unintended outcomes [19]. Hence, this evaluation study complemented the three qualitative studies conducted on PCNs by harvesting diverse outcomes and shedding insights in areas not covered previously [38,39,40].

In addition, it is challenging to design a monitoring and evaluation framework from the start of a complex programme or when the programme evolves with time. Outcome harvesting is therefore suitable for assessing complex programmes where such evaluation frameworks have not been made available from the start.

As the evaluation was data-intensive, the principal investigator had to double up to verify the outcomes when the data should have ideally been verified by a paid external consultant. This was not a major limitation considering that most of the outcomes could be verified by the two primary care experts serving as the key informants for the outcomes.

Outcomes captured was also subjected to recall bias and dependent on participant’s awareness of the outcomes. This bias was mitigated by getting more leaders from the same PCN to participate together and by designing clear and comprehensive thematic outcome categories. Moreover, the iterative processes of clarifying the outcomes with the participants through the survey and follow-up platforms were judged to have succeeded in exhausting the possibilities for outcomes.

Although the inclusion of PCN GPs and patients would corroborate the outcomes better, the scope of PCN outcomes would not have significantly changed by extending the inclusion criteria. As this evaluation was exploratory, follow-up research to further evaluate specific PCN GP- or patient-related outcomes such as perceived facilitators and barriers influencing ancillary service utilisation may be considered.

## 5. Conclusions

PCNs have successfully fulfilled most of the programme aims and objectives by demonstrating wide-ranging outcomes. Hence, the PCN programme will contribute significantly as an integral part of the national Healthier SG ecosystem.

COVID-19 has taught us many valuable lessons, most importantly that ‘trust has been the most critical factor in Singapore’s pandemic response’. As primary and preventive care sectors take on pivotal roles in the paradigm shift to population health in Healthier SG, the same three key thrusts of trust: competence, commitment and transparency, are important fundamentals to fall back on.

First, the evaluation has demonstrated that GP competencies are best harnessed in networks and through a fine balance between a centralised and a ground-up approach. Second, MOH, POA and PCNs have invested much resources to build trust and commitment, which should be leveraged for Healthier SG. Lastly, as all stakeholders strive towards developing transparency, the approach will serve to align all stakeholders to the common goal.

## Data Availability

Data are available on reasonable request. Transcripts will not be shared to protect the anonymity of the participants. Readers who wish to gain access to the data can write to the corresponding author.

## Abbreviations

CDR: Chronic Disease Registry; CHAS: Community Health Assist Scheme; EMR: Electronic Medical Records; FMC: Family Medicine Clinic(s); GAD7: Generalised Anxiety Disorder Assessment; GP: General Practitioner(s); GPII: GP Innovation Initiative; HQ: Headquarter(s); IR(s): Intermediate Result(s); IT: Information technology; M&E: Monitoring and Evaluation; MOH: Ministry of Health; MOHT: MOH Office of Healthcare Transformation; PCN(s): Primary Care Network(s); PHQ9: Patient Health Questionnaire-9; POA: The PCN Oversight Agency.

## Appendix A. Breakdown of Charted Data into Tabular Format

**Table A1:** Summary table of positive and negative outcomes for intermediate results (IRs) ^a^ by year 2018–2021.

Year	Total (+)	Total (−)	IR1 (+)	IR1 (−)	IR2 (+)	IR2 (−)	IR3 (+)	IR3 (−)	IR4 (+)	IR4 (−)
2018	63	−13	37	−10	12	−2	16	−1	13	−1
2019	23	−2	4	−2	3	0	10	0	15	0
2020	28	−4	7	−2	12	0	7	−1	7	−2
2021	20	−3	5	−1	8	0	1	0	9	−2

^a^ Some outcomes have been contributed by more than one IR.

**Table A2:** Summary table of the positive outcomes (*N* = 134) in categories (Cat 1–8) ^a^ and contributed by intermediate results (IRs) ^b^.

IRs	Cat 1	Cat 2	Cat 3	Cat 4	Cat 5	Cat 6	Cat 7	Cat 8	Grand Total
IR1	22	0	2	5	0	8	12	4	53
IR2	9	3	4	9	1	3	2	4	35
IR3	1	0	1	12	8	0	0	12	34
IR4	3	5	2	13	12	1	2	6	44

^a^ Category 1 Network Development; Category 2 Service Provision; Category 3 Clinical Practice; Category 4 Care Delivery; Category 5 Benchmark and Standards; Category 6 Resource Sharing; Category 7 Harnessing Enablers; Category 8 Learning. ^b^ Some outcomes have been contributed to by more than one IR.

**Table A3:** Summary table of the negative outcomes (*N* = 22) in categories (Cat 1, 2, 4, 5 and 6) ^a^ and contributed intermediate results (IRs) ^b^.

IRs	Cat 1	Cat 2	Cat 4	Cat 5	Cat 6	Grand Total
IR1	3	4	1	0	7	15
IR2	1	0	1	0	0	2
IR3	0	0	2	0	0	2
IR4	1	0	2	2	0	5

^a^ Category 1 Network Development; Category 2 Service Provision; Category 4 Care Delivery; Category 5 Benchmark and Standards; Category 6 Resource Sharing. ^b^ Some outcomes have been contributed to by more than one IR.

## Appendix B. Grouped Outcomes

**Table A4:** Matrix of thematic outcome categories ^a^, outcomes were dichotomised using a traffic lights system to highlight positive and negative effects ^b^.

Outcome Category	Key Outcomes Sub-Category	Grouped Outcomes	S/N of Individual Outcome (*N* = Total)
Category 1: Network Development	Governance, resource allocation and involvement of parent organisation	Committed parent organisations have contributed to the development of governance structure, additional resources and cohesion within the network.	4, 5, 6, 25, 71, 72 (*N* = 6)
		The homogeneous network and centralised leadership observed in corporate chain PCNs and their parent organisations have afforded them greater autonomy in the development of strategic plans.	15, 17 (*N* = 2)
		The parent organisation of a PCN had allowed its partner clinic, which had pre-existing affiliations with another PCN, to join the latter. These turn of events have encouraged collegiality amongst all parties involved.	14 (*N* = 1)
	GP onboarding, engagement and cohesion building	The organic GP onboarding observed in corporate chain PCNs and pre-existing GP affiliations in some cluster-led PCNs have resulted in a more seamless GP onboarding and engagements.	13, 19, 25, 26(*N* = 4)
		The onboarding and engagements of GPs who had previously been working in siloes were more resource intensive.	8 (*N* = 1)
		Structured engagements have provided the impetus for network development and ongoing GP support for the development of additional GP competencies. By operating within a safe and conducive space, GPs have been able to transition from siloed practices to a team-based care model.	1, 2, 3, 9, 10, 11, 17, 20, 21, 27, 40, 57, 75, 78, 109, 142, 147 (*N* = 17)
		Corporate chain PCNs have operated from pre-existing smaller GP clusters led by competent GP leads. By decentralising into smaller clusters, these PCNs have been able to customise the provision of GP support, collaborative learning and engagements.	16, 95 (*N* = 2)
	Corporate governance	Some PCN HQ staff have been assigned specific business management roles e.g., finance team. This has helped develop some degree of corporate governance capabilities within the HQs needed for effective strategic planning.	56, 66 (*N* = 2)
	Pre-existing network and infrastructure	Some corporate chain and cluster-led PCNs have been able to leverage on pre-existing infrastructure (manpower, operational framework, IT networks) in the initial set up of their networks.	19, 37, 71, 124, 127 (*N* = 5)
	GP engagement	There is a fine balance between creating a conducive environment and feeding into a dependent culture.	12 (*N* = 1)
	Limited Resource Allocation	Despite the availability of piece-rate subvention, PCN HQ has had to generally operate within the overall subvention framework that was projected from the outset. Such projections lacked the visibility of the increase in workload and impact of COVID.	5 (*N* = 1)
	Cluster Catchment Policy	Cluster-led PCNs have had to contend with the strategic dilemma on the extent to which they should focus on the sector or lateralise the GP catchment. The different catchment policies have contributed to the variations in tertiary care access and coverage.	24, 32, 39 (*N* = 3)
Category 2: Service Provision	Models of Ancillary Service Delivery	All PCN HQs have adopted different ancillary service delivery models (in-house, external and mixed) in order to extend the coverage and increase the utilisation of ancillary services.	18, 29, 30, 31, 33, 36, 37, 62, 74, 76 (*N* = 10)
		Most PCNs that have developed in-house capabilities have had to leverage on external providers for ancillary services or collaborate with DRP machine suppliers initially.	30, 107, 114 (*N* = 3)
		Clustering of in-house ancillary service capabilities has enabled PCN HQs to decentralise and customise service delivery, thereby extending coverage and improving utilisation.	18, 29, 36(*N* = 3)
		One PCN has embarked on a MOHT GPII to consolidate existing network of effective and accessible private optometrists/opticians in order to deliver DRP services.	82 (*N* = 1)
	Ground-up Initiatives	A PCN collaborated with a voluntary welfare organisation in the ad hoc delivery of ancillary services to a migrant population. In the collaboration, three migrant workers were found to have diabetic retinopathy, one of whom needed two laser treatments.	110 (*N* = 1)
		Ground-up PCN GP initiatives through the sharing of best practices and collaborations have resulted in new PCN-wide services e.g., ambulatory telemetry, counselling	35, 41, 47, 130 (*N* = 4)
	Financial Access	Patients who have pre-existing rapport with GPs are increasingly channelled away to polyclinics due to the financial gradient between GP clinics and the polyclinics. This is despite availing CHAS subsidies for use in GP clinics.	34 (*N* = 1)
	Internal referral system	The internal referral system to tertiary care within an organisation has resulted in the under-development of mental health care delivery amongst its PCN GPs.	38 (*N* = 1)
Category 3: Clinical Practice	Mental Health Care Initiatives	Some PCN HQs have collaborated with external stakeholders to provide mental health GP accreditation training and set up in-house services for low-intensity psychotherapy. Some PCNs have operationalised mental health screening with PHQ2, PHQ9 and GAD7.	41, 42, 44, 46, 50, 55, 116, 130, 147, 152 (*N* = 10)
	Secondary screening	The chronic disease registry (CDR) has been utilised to screen for pre-diabetes using the bone mass index (BMI) values for non-diabetic patients and for osteoporosis using Osteoporosis Self-Assessment Tool for Asians (OSTA) scores.	43, 45 (*N* = 2)
	Clinical Audits	In one PCN, an expert committee was set up in the selection of external providers for DRP services. The DRP reports have also been vetted by the ophthalmologists from the parent organisation.	49 (*N* = 1)
Category 4: Care Delivery (Operations, Logistics, Marketing)	One-Stop Ancillary Services	In order to address the clinic space and operational constraints, one-stop ancillary service has been provided in nearby larger clinics, by utilising mobile vans, by equipping and training HQ/clinic staff and by operating in the clinic during non-clinic hours.	18, 30, 31, 52, 59, 62, 80 (*N* = 7)
	Nurse Counselling (NC)	Structured workflows, tele-counselling, increasing availability of nurse counsellors and bundle pricing have increased NC uptake.	53, 54, 58, 69, 124 (*N* = 5)
	Administrative support	Programme managers and primary care coordinators have been effective in engaging GPs/clinic staff, providing support for data management and quality improvement initiatives.	10, 30, 57, 75, 77, 85 (*N* = 6)
	Platform for COVID initiatives	Using their effective networks for expedient cascade of services/directives, PCNs were able to rally public health preparedness clinics in urgent capacity building and dissemination of resources e.g., for COVID responses.	51, 60, 61, 79, 81 (*N* = 5)
	Ancillary service delivery	A PCN GP has chosen to refer his patients to external providers instead of utilising the in-house ancillary services provided in a nearby larger clinic as he was worried about losing patients to that clinic.	52 (*N* = 1)
	COVID impact on chronic disease management	The increase in medication delivery and tele-consults due to COVID have resulted in a drop in physical clinic attendances, thereby disrupting proper chronic disease care delivery and adherence to CDR processes.	61 (*N* = 1)
	Limited Resources for ancillary service delivery	By sharing a roving team with 1 DRP machine between 10 satellite clinics, each clinic was only able to provide ancillary services for up to two months in the year.	70 (*N* = 1)
Category 5: Benchmarks and Standards	Data Management (data collection, transfer, maintaining data integrity and analysis)	Some PCN HQs, GPs and clinic staff have incorporated structured data management processes to meet the CDR requirements and Macro Excel revision.	64, 77, 92(*N* = 3)
		Most PCN HQ staff have learnt to manage data whilst on the job. Only three PCNs recruited staff with data analysis background.	87, 89, 96 (*N* = 3)
		Corporate Chain PCNs have been able to semi-automate data management processes by modifying pre-existing EMR systems and integrating EMRs with in-house laboratory and imaging services.	112, 125, 127, 128, 129(*N* = 5)
		A cluster-led PCN used its pre-existing network EMR to seamlessly transition into PCN.	96 (*N* = 1)
		Different IT platforms for secured storage and transfer were utilised for data management, one of which was acquired with productivity solutions grant.	118, 123 (*N* = 2)
		One PCN collaborated directly with an EMR provider to automate upstream data collection and data migration.	98 (*N* = 1)
	Gap Analysis and Quality Improvements	All PCNs have conducted gap analyses, which have been regularly communicated with GPs and clinic staff e.g., during the mid-year and end-year reviews with GPs. The regularity of the reviews have developed in the GPs a sense of ownership for the data and quality improvement initiatives.	75, 85, 86, 88, 91, 93, 95, 99, 139, 149 (*N* = 10)
		Some PCNs have analysed the trends for clinical and operational gaps using the CDR data before discussing quality improvement initiatives and best practices.	48, 91, 93, 95, 96 (*N* = 5)
	Data Presentation & Dissemination	Several PCNs have designed their individual primary care dashboards. These dashboards have provided visualisation of the clinic’s performance year-on-year and with benchmarking.	94, 96, 135 (*N* = 3)
	Process Indicators	By managing chronic diseases using the clinical guidelines embedded in the CDR, ‘the unintended outcome is to further entrench the disease-defining practice rather than on personhood-base practice’.	90 (*N* = 1)
	Pay-for-Performance framework	The focus of the Care Plus Fee criteria on diabetes and complex chronic conditions can potentially result in bias as simple chronic conditions e.g., isolated hypertension or hyperlipidaemia] may be omitted from the CDR.	97 (*N* = 1)
	Administrative burden of CDR	The GPs from a couple of clinics within a PCN have chosen not to contribute to the CDR, citing administrative burden. At one point, the GPs have threatened to drop out of the PCN.	2 (*N* = 1)
Category 6: Resource Sharing and Integrated Care	Right-siting	With the ongoing support from their respective PCNs, two FMCs have been able to sustain significant volume of decanted chronic patients. Synergistic partnerships, comparable care/services, drugs at similar subsidised rates were the attributes demonstrated by these programmes.	67, 68, 105 (*N* = 3)
	GP+ Co-operative	Some PCN leaders and GPs have set up a co-operative of doctors with the aim of transforming healthcare delivery and benchmark quality in primary care.	102, 115 (*N* = 2)
	Medication Access	Two PCNs have collaborated with hospitals to avail subsidised medications to decanted patients. However, these collaborations are resource intensive, requiring substantial logistical coordination and administration.	55, 84 (*N* = 2)
	Right-siting	The economies of scale from banding together as a PCN or as a group of PCNs have not been able to reduce the cost of medications sufficiently to match the rates polyclinics enjoy.	63, 104 (*N* = 2)
		Most right-siting programmes were perceived to have encountered challenges with scaling.	68, 100, 101, 103, 106, 113 (*N* = 6)
		The GPs in one PCN, who had been well supported by their PCN HQ and self-sufficient with their own pool of chronic disease patient load, chose not to subscribe to right-siting programmes.	108 (*N* = 1)
Category 7: Harnessing Enablers	EMR adoption	The majority of GPs in one PCN had been using their EMRs only for administrative functions. EMR champions and early adopters were instrumental in improving the utilisation for the full EMR within that PCN using diffusion of innovation.	121 (*N* = 1)
	Patient Engagement Apps	Two PCNs have collaborated with MOHT and a parent organisation to design and roll out individual patient engagement apps to facilitate care delivery and encourage patient ownership in chronic disease management.	122, 126 (*N* = 2)
	Pay-for-Performance framework	With the support of the parent organisation, a cluster-led PCN has incentivised its GPs for every patient who has achieved the composite cut-off of six out of eight process indicators.	119 (*N* = 1)
Category 8: Learning initiatives	Collaborative learning	Regular team-based activities and communication (e.g., WhatsApp group chats) amongst GPs have encouraged collaborative learning and established good practices.	133, 136, 140, 144, 148, 150, 151 (*N* = 7)
		During an appreciation lunch, one PCN ‘celebrated’ the efforts and good performances of its GPs in driving clinical indicator improvements.	138 (*N* = 1)
	Training	In addition to the training provided by the PCN oversight agency, PCN HQ provided its staff with additional training in order to develop relevant competencies.	131, 132, 134, 137, 141, 146(*N* = 6)
		One of the bigger PCNs have allocated substantial resources in formalising training of clinic staff in order to scale the operations and clinical standards expected of the PCN clinics.	143 (*N* = 1)

^a^ Some outcomes have been classified in more than one category. ^b^ Traffic light classification highlighting positive outcomes in green and negative ones in red; outcomes with scaling challenges are highlighted in orange.
